# CIRSE standards of practice on gynaecological and obstetric haemorrhage

**DOI:** 10.1186/s42155-020-00174-7

**Published:** 2020-11-27

**Authors:** Thomas Rand, Rafiuddin Patel, Wolfgang Magerle, Raman Uberoi

**Affiliations:** 1 Institute for Interventional and Diagnostic Radiology, Klinik Floridsdorf, Brünnerstr.68, 1210 Vienna, Austria; 2 Scientific research in diagnostics and interventional radiology, Karl Landsteiner Society, St. Pölten, Austria; 3 John Radcliffe Hospital, Oxford University Hospitals NHS Trust, Oxford, UK; 4 Klinik Hietzing, Vienna, Austria

**Keywords:** Obstetric haemorrhage embolisation, Uterine artery embolisation, Postpartum haemorrhage, Trauma

## Abstract

This CIRSE Standards of Practice document provides best practices for obstetric haemorrhage embolisation (OHE) in the management of postpartum haemorrhage (PPH). The document is aimed at interventional radiologists involved in treating postpartum haemorrhage, and has been developed by a writing group established by the CIRSE Standards of Practice Committee.

CIRSE Standards of Practice documents are not clinical practice guidelines and do not intend to impose a standard of care, rather provide reasonable approaches to and best practices for specific interventional radiology treatments and techniques.

## Background

### Introduction

Genital bleeding is a major cause of morbidity and mortality in women, particularly during childbearing years. Severe postpartum haemorrhage (PPH) plays a prominent role as a cause of maternal death and accounts for 25% of maternal deaths worldwide. In developed countries, it is estimated that there are 9–17 maternal deaths related to PPH per every 100,000 deliveries, as compared to 400 deaths per 100,000 deliveries worldwide (World Health Organization, UNICEF, World Bank, and United Nations Population Fund 2007; Khan et al. 2006).

PPH is divided into primary haemorrhage, which indicates excessive bleeding from the genital tract of > 500 ml in the first 24 h after a vaginal delivery, and secondary haemorrhage, which occurs after the first 24 h up to 6 weeks after the birth (Lopera et al. 2013; Briley et al. 2014; Gonsalves and Belli 2010).

The most common causes of primary PPH include uterine atony, when the normal myometrium fails to contract after delivery of the placenta, genital tract injuries such as perineal or vaginal lacerations, uterine rupture or inversion, placental implantation abnormalities, pseudo-aneurysms and congenital or acquired coagulation disorders (Gonsalves and Belli 2010).

Secondary PPH is mainly related to retained products of gestation or infection. Other conditions resulting in significant urogenital haemorrhage include gynaecological cancers, post-operative bleeding, trauma, arteriovenous malformations (AVMs), such as congenital and acquired AVMs and arteriovenous (AV) fistulas (Soyer et al. 2015; Mihmanli et al. 2001; O’Brien et al. 2006; Katz et al. 2012; Josephs 2008; Field et al. 2016).

Initial management of PPH with fluid resuscitation, correction of any coagulopathy, removal of retained placental tissue, uterotonic drugs and balloon tamponade will stop bleeding in 85% of patients. Where these measures fail, for many years, internal iliac artery ligation has been used as a potentially effective means of controlling pelvic haemorrhage (Burchell 1968; Chattopadhyay et al. 1990; Evans and McShane 1985; Katz et al. 2012). The effectiveness of internal iliac artery ligation results from reduced pelvic blood flow and subsequent haemostasis. However, this may be successful in < 50% of the cases, potentially leading to a high rate of hysterectomy up to 50% (Clark et al. 1985, B-Lynch et al. 2012).

In 1969, Nusbaum demonstrated that arterial bleeding might be controlled by selectively infusing a vasoconstrictor into the affected artery (Nusbaum et al. 1969). His technique started the evolution of transcatheter selective obstetric haemorrhage embolisation (OHE) in otherwise untreatable bleeding. Athanasoulis et al. reported in 1976 the first series of gynaecological bleeding to be treated with embolisation (Athanasoulis et al. 1976). Subsequently, Brown and Heaston used this technique to treat PPH in 1979 (Brown et al. 1979; Heaston et al. 1979). From this time, with the evolution of endovascular techniques, OHE has emerged as a highly effective percutaneous technique for controlling gynaecologic and obstetric haemorrhage.

Uterine rupture and eversion may cause severe blood loss. Although its treatment is managed primarily by surgery, there are endovascular options stated (Hofmeyr et al. 2005; Lopera et al. 2013; Gonsalves and Belli 2010).

Table 1 provides a summary of key recommendations for gynaecological and obstetric haemorrhage.

**Table 1:** Summary of key recommendations

Recommendation	
**Indications**	For genital bleeding following vaginal or caesarean delivery or due to surgical complications, including post hysterectomy, OHE should be considered, particularly if bleeding is due to spontaneous pseudoaneurysm rupture.
	Prophylactic balloon catheter occlusions and/or uterine embolisation can be used for patients undergoing planned hysterectomy, as well as for those who wish to have conservative management with uterine sparing techniques.
	If all medical measures and surgical interventions are unsuccessful, uterine artery embolisation (UAE) should be performed before hysterectomy if the woman is haemodynamically stable enough to be moved and there is an embolisation service available nearby. Embolisation can also be undertaken in a theatre environment where there is access to hybrid theatre facilities.
**Contraindications**	Uterine rupture and eversion should be treated with surgery, however there are no absolute contraindications regarding OHE.
**Imaging**	In a slower intermittent bleed, and/or if relevant for the procedure, ultrasound, CT and MR may be helpful prior to proceeding to catheter angiography.
	Triple-phase CT protocols are recommended, with an unenhanced scan followed by arterial phase (30 s) and a delayed portal venous phase (60–70 s).
**Procedure**	In PPH, arterial embolisation is preferentially performed with non-permanent embolic material i.e. resorbable pledgets in both uterine arteries.

### Definitions

#### PPH

Blood loss of > 500 ml following vaginal delivery or > 1000 ml following caesarean section. (Lopera et al. 2013).

#### Severe haemorrhage

Any amount of bleeding, that, if not replaced, could cause shock or death in the mother.

#### Primary PPH

Indicates excessive bleeding from the genital tract of 500 ml or more within the first 24 h after a vaginal delivery (Briley et al. 2014).

#### Secondary PPH

Bleeding that occurs after the first 24 h and up to 6 weeks after the birth (Lopera et al. 2013; Gonsalves and Belli 2010).

#### Gynaecological bleeding

Bleeding from gynaecological cancer, AVMs, AV fistulas, and intractable post-operative haemorrhage (Mihmanli et al. 2001; O’Brien et al. 2006; Field et al. 2016; Sentilhes et al. 2016; Briley et al. 2014; Sheldon et al. 2014; Pelage et al. 1998; Pelage et al. 1999).

#### Clinical success of OHE

Defined as stopping the haemorrhage, regardless of number of OHE procedures, with no subsequent surgical procedure due to persistent bleeding.

#### Anatomical blood supply

**Fig. 1:**
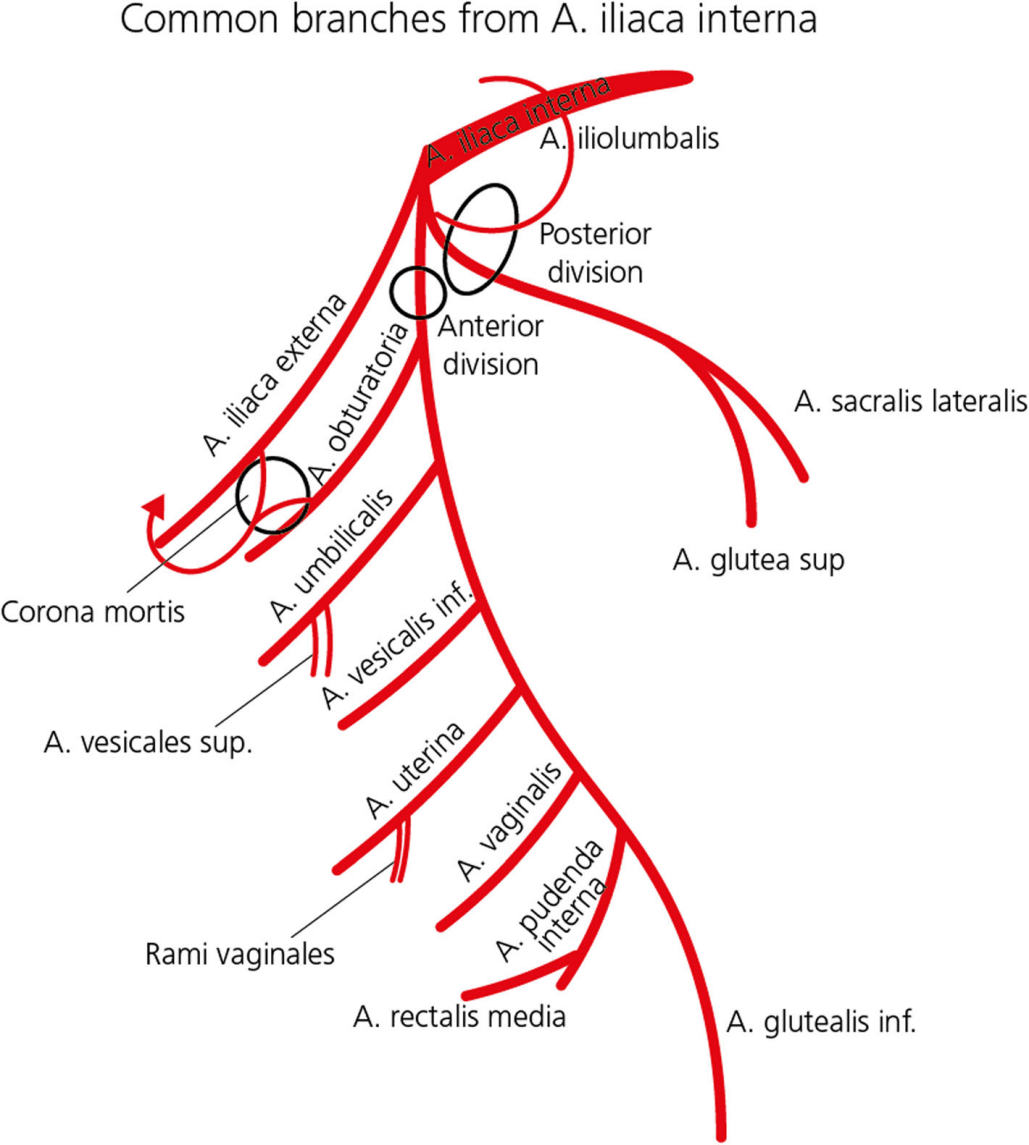
Internal iliac artery and branches: overview

The internal iliac artery (IIA) divides into a posterior and an anterior division, the latter giving rise to several parietal and visceral branches (Fig. 1). The visceral branches are the key vessels for OHEand include uterine, vaginal, and internal pudendal arteries. Parietal branches of the anterior division of the IIA include obturator and inferior gluteal arteries. Moreover, various anastomoses exist between the IIA, mesenteric arteries and external iliac artery branches as well as anastomoses between the anterior and posterior division arteries (Ouyang et al. 2012; Matson et al. 2000).

**Fig. 2:**
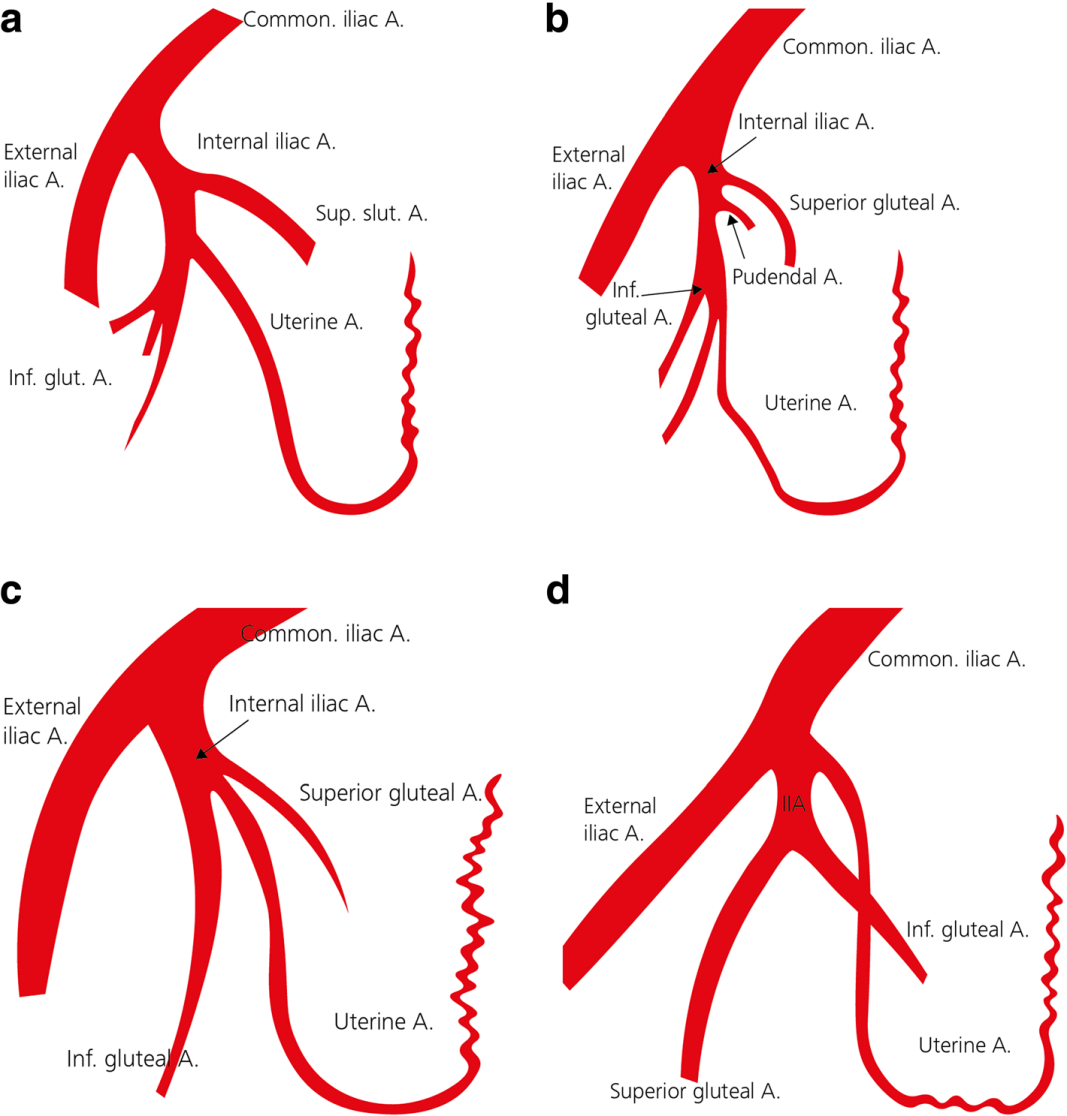
**a-d** Anatomic variations of normal uterine artery. **a** Uterine artery as 1st branch from inferior gluteal art (type I). **b** Uterine artery as 2nd or 3rd branch from inferior gluteal art (type II). **c** Inferior gluteal, superior gluteal artery, and the uterine artery all arise at the same level (trifurcation) (type III). **d** Uterine artery arises proximal to the origin of the inferior gluteal & superior gluteal arteries (type IV)

The uterine artery (UA) is most relevant to OHE in the setting of obstetric and gynaecological haemorrhage. Depending on its level of origin, the UA is classified in to one of four types (Fig. 2a-d). The course of the uterine artery typically includes a hairpin curve where the artery passes through the cardinal ligament at the base of the broad ligament (Albulescu et al. 2014; Farrer-Brown et al. 1970; Razavi et al. 2002).

**Fig. 3:**
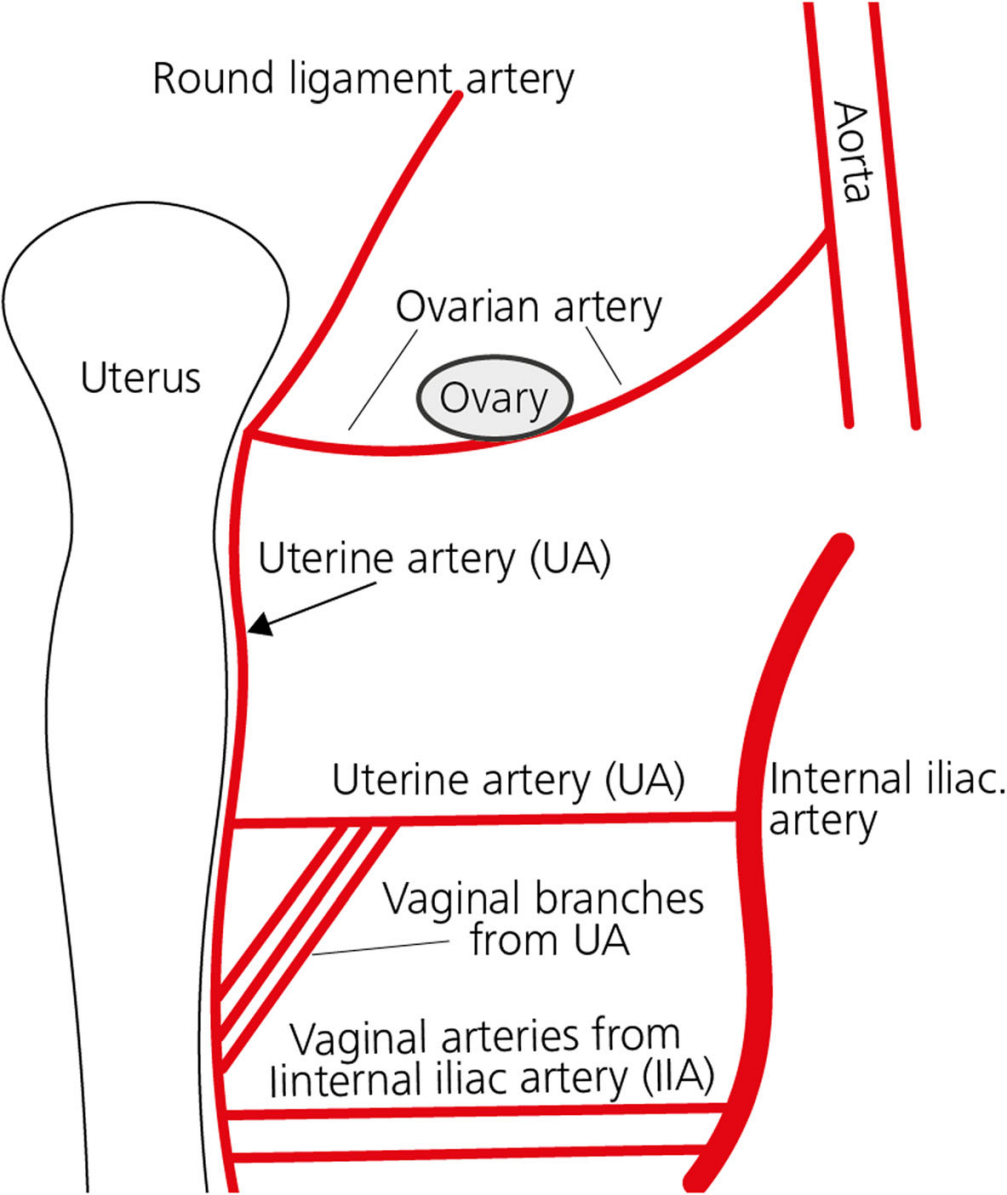
Arterial Anastomotic system blood supply of the uterine artery (UA)

**Fig. 4:**
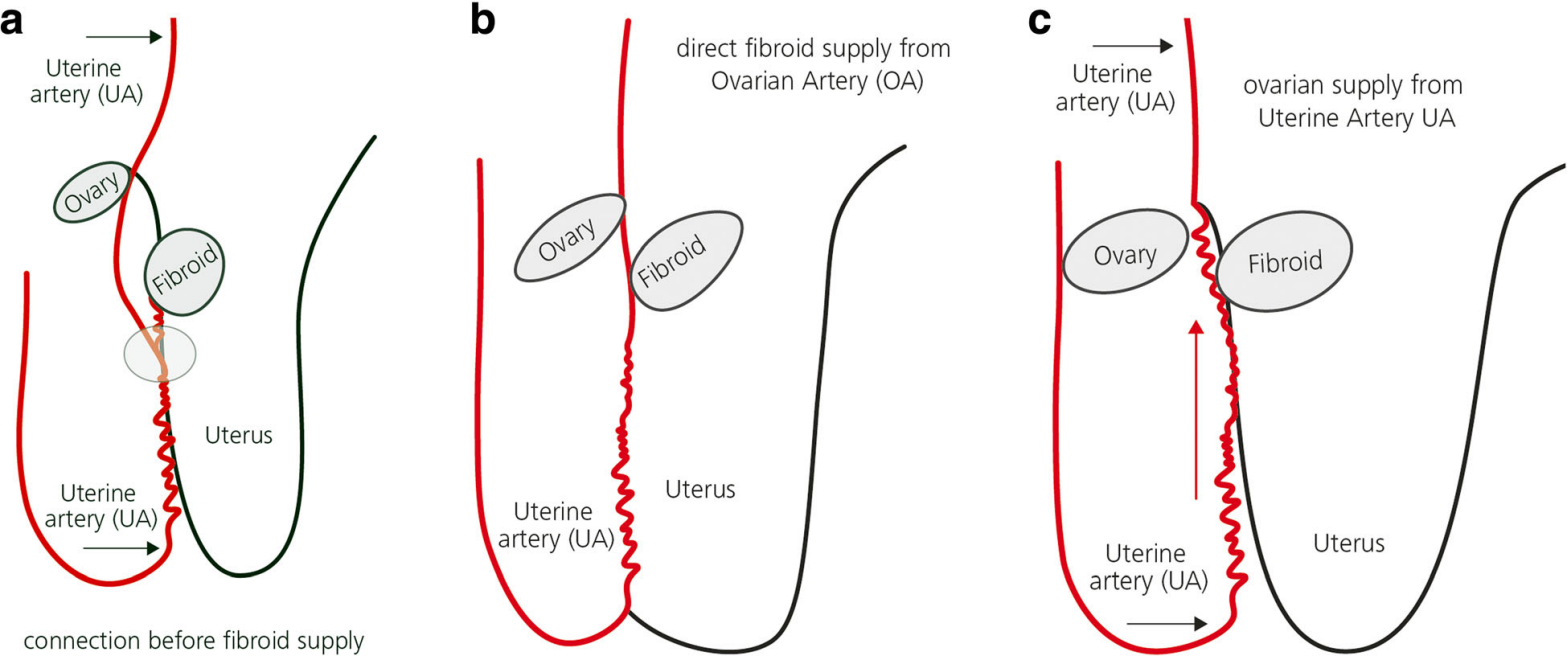
**a-c** Anastomotic blood supply in case of fibroids. **a** Connection before fibroid supply. **b** Direct fibroid supply from ovarian artery (OA). **c** Ovarian supply from uterine artery (UA)

Further key arteries are vaginal arteries stemming from the anterior division of the IIA below the origin of the UA or from the UA itself. Cervicovaginal branches originating from the uterine arteries may also supply the cervix. Branches from the inferior vesical artery supply the middle portion of the vagina. The internal pudendal artery supplies the lower cervical portion, whereas the posterior portion is generally supplied by the middle rectal artery. There is a continuous arcade on the lateral borders of the vagina, uterus and adnexa. The cervicovaginal arteries arise from the uterine artery (Palacios Jaraquemada et al. 2007). Anastomoses between the uterine and the ovarian arteries are commonly present (Figs. 3 and 4 ) (Razavi et al. 2002).

Important anatomical variations also need to be considered, e.g. a persistent sciatic artery (Brantley et al. 1993), originating from the anterior IIA division and a “corona mortis”, Latin for “crown of death”, a common variant vascular anastomosis between the external iliac artery or deep inferior epigastric artery with the obturator artery present in a third of patients (Smith et al. 2009). AVMs and AV fistulas in particular are more likely to have variations in arterial blood supply. They most frequently arise from internal pudendal arteries, cervical branches of the uterine artery, or from vaginal arteries after vaginal birth and from uterine arteries after caesarean section (O’Brien et al. 2006).

## Methods

The writing group, which was established by the CIRSE Standards of Practice Committee, consisted of 4 clinicians with internationally recognised expertise in gynaecological and obstetric haemorrhage. The writing group reviewed existing literature on gynaecological and obstetric haemorrhage and OHE, performing a pragmatic evidence search using PubMed to search for relevant publications in the English language.

## Treatment

### Pre-treatment imaging

If conservative treatment options are exhausted, uterine artery embolisation (UAE) should be performed if the woman is haemodynamically stable enough to be moved and there is an embolisation service locally available.

OHE does not require general anaesthesia, but close monitoring and support to maintain blood pressure requires the presence of an anaesthetic team. Following a right-sided unifemoral approach using a 4-5F-sheath, a selective internal iliac digital subtraction angiography is performed to determine anatomy and to locate possible extravasation of contrast agent. A contralateral internal iliac angiography can be performed initially, and then a selective examination of the uterine artery can be subsequently attempted in almost all cases. The ipsilateral internal iliac artery and uterine artery can also be catheterised with the same catheter via the same puncture site. Oblique views and distal superselective injections for other anastomotic vessels, such as the vaginal branches, may be required to identify the arterial bleeding site.

In a slower intermittent bleed ultrasound, CT and MR may be helpful prior to proceeding to catheter angiography (Sierra et al. 2012; Lee et al. 2010; Cano Alonso et al. 2009; Vasanawala et al. 2006).

#### Ultrasound

Transabdominal and/or transvaginal ultrasound may be performed at the bedside. Ultrasound may show abdominal fluid and retained products. Doppler studies may also help assess any AVMs or AV fistulas (O’Brien et al. 2006), however, they play a minor role in acute haemorrhage.

#### CT

If relevant for the interventional procedure, Triple-phase CT protocols are recommended, with an unenhanced scan followed by arterial phase (30 s) and a delayed portal venous phase (60–70 s). Comparing the unenhanced scan with the contrast-enhanced scan will demonstrate any pre-existing hyperattenuation to help differentiate areas of calcifications, hematoma, or postoperative material from extravasation during the contrast-enhanced phase. However, if diagnosis and management is clear, patients may also directly proceed to angio and intervention.

The delayed portal venous phase imaging helps to differentiate arterial haemorrhage from engorged vessels, venous bleeding or pseudoaneurysms. Low-dose CT protocols, which do not compromise diagnostic certainty, should be utilised where possible to reduce the potential radiation dose from a triple-phase study.

CT can be particularly useful for extra-uterine sources of bleeding, such as rectus sheath hematoma, and may also detect AV fistulas and/or uterine AVMs, direct arterial injuries, dehiscence of the caesarean scar, bladder flap hematoma, para-vaginal hematomas, potential arterial collateral vessels, myometrial disruption and hemoperitoneum (Takeda et al. 2014).

#### MRI

MR-Angiography (MR-A) may give similar information to CT and can be extremely useful in diagnosing abnormal placentation during gestation (Wang et al. 2017). However, MR-A is uncommonly used in an acute setting.

### Indications for treatment

#### PPH

**Fig. 5:**
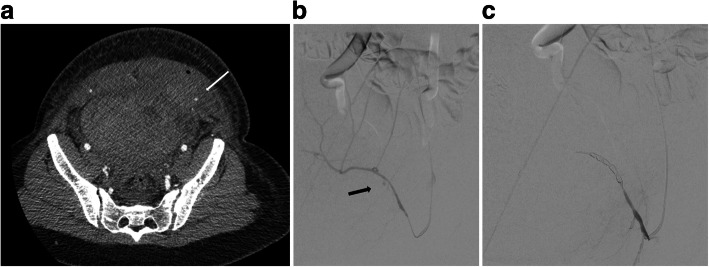
**a-c** Haemorrhage and embolisation due to PSA bleeding. **a** CT shows abdominal wall haematoma with small pseudoaneurysm. **b** Angiography from a contra-lateral approach with 4F cobra catheter and microcatheter use, confirms small false aneurysm arising from the inferior epigastric artery. **c** superselective embolisation with multiple 2 and 3 mm micro coils

For genital bleeding following vaginal or caesarean delivery, or due to surgical complications including post hysterectomy (Cheng et al. 2017; Lee et al. 2012a; Fu et al. 2018), OHE should be considered, particularly if bleeding is due to spontaneous pseudoaneurysm rupture (Fig. 5) (Gonsalves and Belli 2010).

#### Placenta previa, accreta and percreta

Placenta previa, placenta accreta, placenta percreta and vasa previa are important causes of bleeding in the second half of pregnancy and in labour (Oyelese and Smulian 2006).

**Fig. 6:**
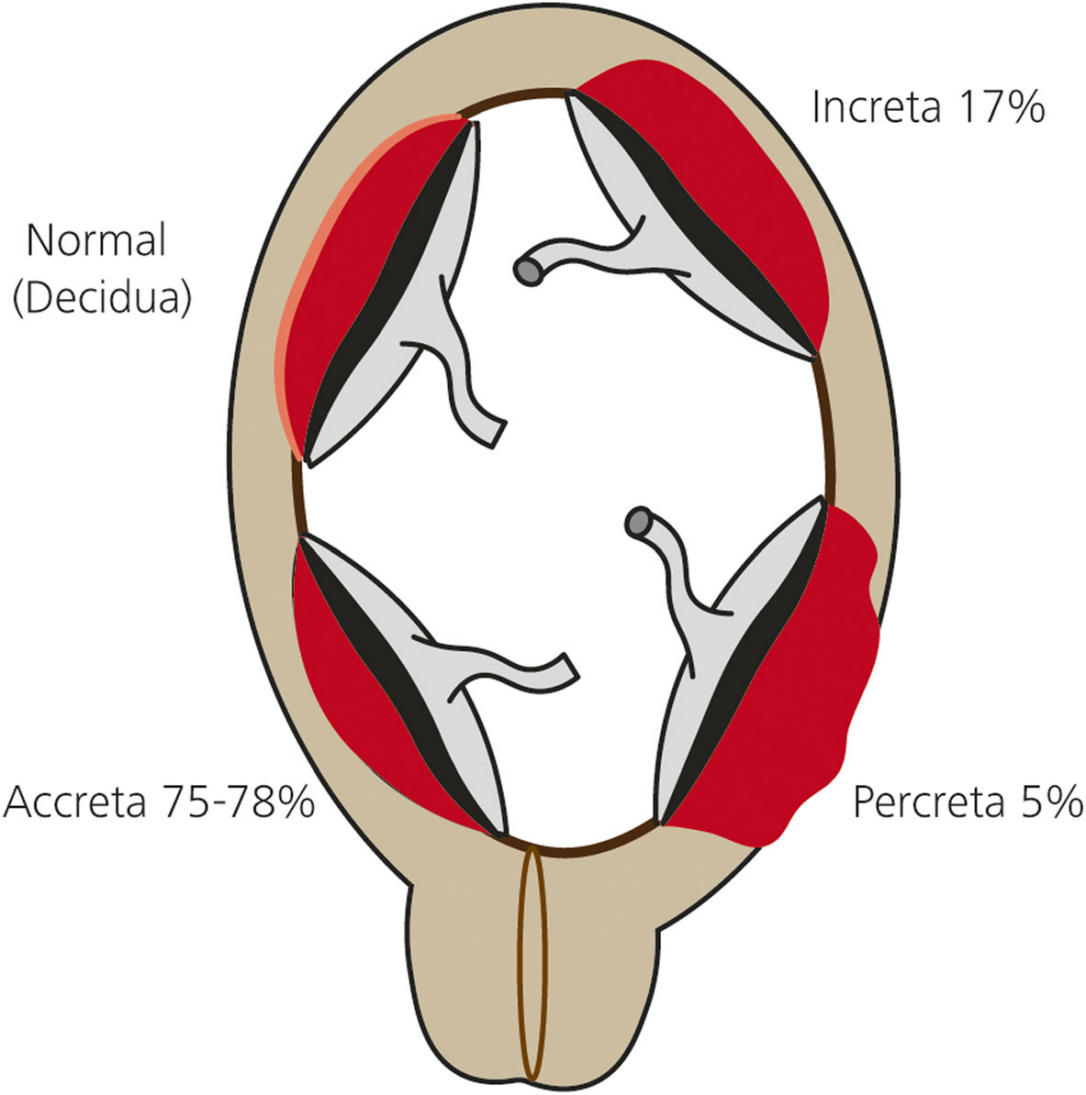
Model figures of placenta accrete, increta, percreta

Abnormal placental implantation is caused by invasion of the chorionic villi through the decidual basal layer into the myometrium (Kaufman and Tadros 2018). Placenta accreta occurs when the chorionic villi attach to the uterine myometrium only, and placenta increta refers to partial invasion of the chorionic villi into the muscle of the myometrium. Placenta percreta is the most severe form, and occurs when there is a chorionic invasion throughout the myometrium into the serosa (Fig. 6). There is a strong association with caesarean section and the incidence is increasing worldwide (Granfors M et al. 2020). Endovascular interventions have been applied in the form of prophylactic balloon catheter occlusions and /or uterine embolisation and are used for patients undergoing planned hysterectomy, as well as for those who wish to have conservative management with uterine sparing techniques (Ojala et al. 2005).

In acute causes of massive bleeding the management is similar to that of PPH (Kaufman and Tadros 2018; DeMeritt et al. 2018; Shahin and Pang 2018; Fitzpatrick et al. 2014; Sentilhes et al. 2013).

#### Gynaecological haemorrhage

**Fig. 7:**
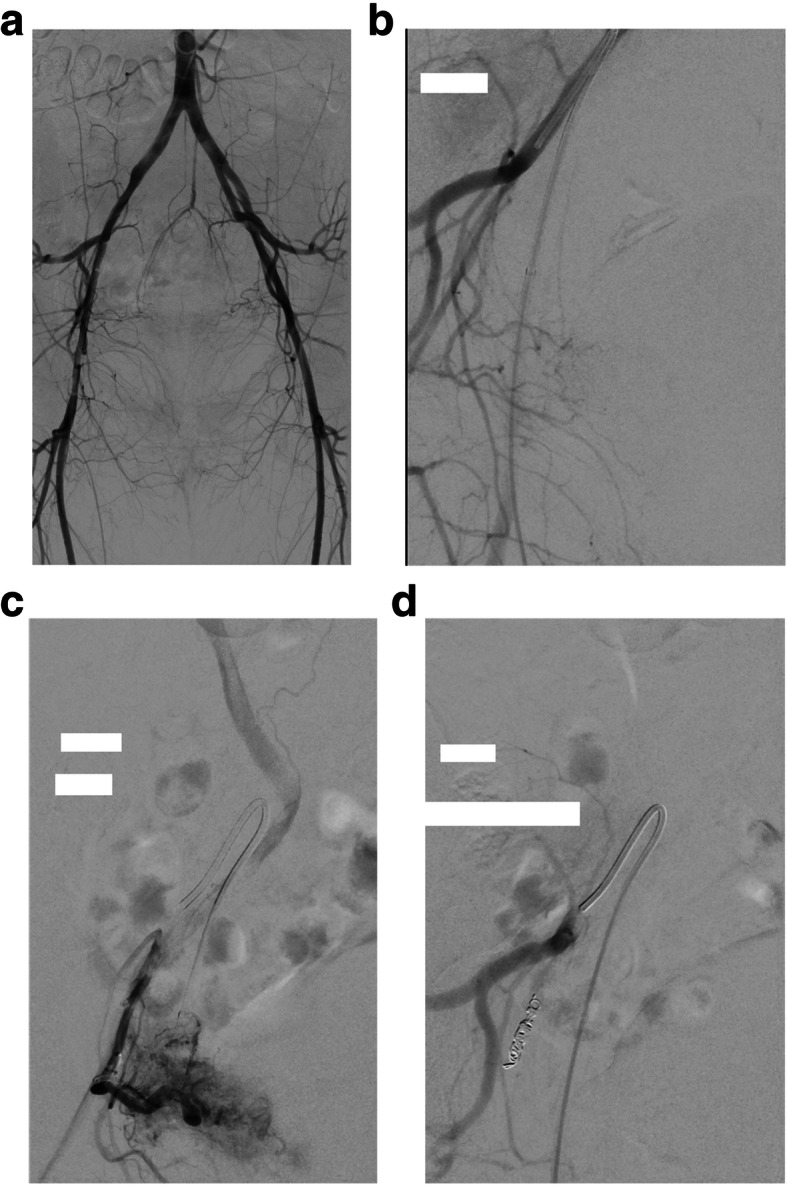
**a-d** Embolisation of postoperative bleeding in malignancy. **a** Angiographic overviews. **b** selective embolisation of the right IAA. **c** Superselective catheterisation of the uterine artery and proof of the bleeding source. **d** superselective embolisation with coils

Commonly, gynaecological malignancies have been managed surgically or by radiation therapy. However, haemorrhage due to advanced-stage disease can be hard to control by surgical means, and embolisation is often preferable (Fig. 7) (Mihmanli et al. 2001; Katz et al. 2012; Field et al. 2016; Pisco et al. 1989; Gmelin et al. 1989).

In cases of tumour invasion of larger arteries such as the external iliac artery, this may also result in major retroperitoneal haemorrhage, and OHE has become the first line treatment (Katz et al. 2012; Pisco et al. 1989).

Pseudoaneurysms may be seen with or without PPH. Uterine AVMs and AV fistulas may be congenital or acquired and, if acquired, usually post-instrumentation or traumatically. Malignancies might also appear in AVMs (O’Brien et al. 2006; Ghai et al. 2003).

Congenital AVMs are more likely to have an arterial blood supply. They most frequently arise from internal pudendal arteries, cervical branches of the uterine artery, or from vaginal arteries after vaginal birth and from uterine arteries after caesarean section (Calligaro et al. 1992).

Both, pseudoaneurysms and AVMs can be treated successfully using OHE. Bilateral embolisation is generally essential, due to the cross-filling of larger arteries of the pelvis.

### Contraindications

Uterine rupture and eversion should be treated with surgery, however there are no absolute contraindications regarding OHE (Gonsalves and Belli 2010; Tourné et al. 2003).

Relative contraindications may appear with the presence of arterial anomalies, and selective embolisation cannot be performed. Relative contraindications to any endovascular intervention include coagulopathy, contrast material allergy and impaired renal function.

### Patient preparation

The CIRSE checklist is a vital tool to ensure necessary safety checks have been performed prior to commencing intervention (Lee et al. 2012a, 2012b). Ideally, OHE should be performed at least 30 min after uterotonic medication administration, because these drugs can induce spasm of the uterine arteries and may make angiographic assessment and treatment more difficult. Facilities for conscious sedation and analgesia are recommended for all procedures.

### Procedural features

#### Equipment specification

OHE should be performed in an angiographic suite with digital subtraction or in an operating room with a similar standard of angiographic equipment. Pulsed fluoroscopy, filtration and optimal technical use of equipment reduces radiation exposure.

When embolisation is performed in a theatre, a pre-prepared ‘emergency haemorrhage control box’ stocked with key equipment which can be easily transported to the theatre is recommended. Specific equipment will vary with operator preferences; examples are given below.

*Standard equipment required includes the following*:

- 4-6F sheaths, including crossover sheaths
- Occlusion balloons
- 4-5F catheters such as Cobra, SOS Omni, RIM Sidewinder, Simmons, etc.
- Micro-catheters
- 0,035``, 0.018`` and 0.014`` guide-wires
- Embolic materials, such as particles (250 mm and larger), Gelfoam, coils, plugs or liquid embolics (Cyanoacrylate, Onyx, Squid), and Spongostan.
- Percutaneous arterial puncture closure devices

#### Embolic materials

Embolic materials can be permanent or temporary. Permanent occlusion is generally used for progressive disease, particularly for bleeding from tumour sites. Temporary embolic agents are more appropriate for self-limiting processes that may heal, such as PPH.

Absorbable gelatine sponges (Gelfoam) contain a water insoluble gelatine that allows vessel recanalisation within several weeks. As absorbable gelatine sponge and particulate agents are not radiopaque, they must be injected with iodinated contrast material.

Permanent embolic materials include microspheres, coils, plugs and liquid polymers. They act by mechanical occlusion and may also activate thrombin formation. Embolic agents are administered until stasis or near stasis is achieved, however dosage must be individually adjusted and should never be overdone.

Permanent liquid embolic agents such as Cyanoacrylate or Onyx might be useful when a catheter cannot be passed to a sufficient distal location. However, time of polymerisation and liquid must be adjusted correctly and appropriate experience is needed (Cheng et al. 2017; Lee et al. 2012a; Fu et al. 2018).

### Techniques

In cases of PPH, active extravasation on digital subtraction angiography (DSA) is a relatively infrequent finding, with detection rates between 21% and 52%. Absence of extravasation is particularly common in the presence of uterine atony. The rate of bleeding required for angiographic detection is 1-2 ml/min, and might be too small to detect in cases of uterine atony (Ganeshan et al. 2010).

Femoral artery access is obtained using a standard Seldinger technique with placement of an appropriately sized sheath (usually 4- or 5F). Bilateral access for embolisation of both uterine arteries simultaneously may save time and radiation. However, unilateral femoral arterial access is commonly used. Following selective and super-selective pelvic arteriography using a 4F or 5F selective catheter (Cobra, Multi Purpose), the uterine artery is cannulated using a 4F- or 5F selective catheter or by a microcatheter (<3F). The use of a microcatheter is generally recommended, as it will prevent spasm and allow more precise distal embolic placement. Arterial spasm often accompanies PPH. If spasm occurs, spasmolytics, such as Nitroglycerin, may be given as a short-acting, safe, effective vasodilator (100–200 Micrograms). However, catheter retrieval and waiting might resolve the problem as well (Gonsalves and Belli 2010; Soyer et al. 2015; Katz et al. 2012; Pelage et al. 1998; Cheng et al. 2017; Lee et al. 2012a; Fu et al. 2018).

For active extravasation the most common source of bleeding is the distal branches of the uterine artery, followed by vaginal arteries. If a bleeding point is found, then super-selective catheterisation and embolisation of the appropriate artery and collateral vessels is performed. If no bleeding site is identified, empiric embolisation of bilateral uterine arteries or the anterior division of the internal iliac artery should be performed, particularly in acute PPH.

When performing OHE, it is important to look for atypical branches prior to embolisation. If the target artery can safely be catheterised, and if reflux or non-target embolisation via anatomic variations can be avoided, OHE can be started.

In PPH, arterial embolisation is preferentially performed with non-permanent embolic material i.e. resorbable gelatine sponge pledgets in both uterine arteries (Hwang et al. 2013). An absorbable gelatine sponge, such as Gelfoam is cut to desired size, and made up into slurry using a mixture of contrast and solution of sodium chloride before being injected intra-arterially as a non-permanent embolic agent to the site of bleeding (Camacho et al. 2019). The procedure should be finished with a flush aortogram at the level of the renal arteries to exclude haemorrhage from vessels not arising from the IIA, such as the ovarian and inferior epigastric arteries.

In severe haemorrhage, angiographic balloon catheters may be required at the outset to achieve haemodynamic control via occlusion at the level of the internal iliac, common iliac arteries or the aorta. Temporary balloon occlusion of the IIA’s before caesarean section has also been advocated to diminish blood loss in cases of abnormal placentation, and can be combined with embolization (Kaufman and Tadros 2018). There is a wide variation in techniques utilised for this approach, with mixed results, and a great degree of controversy remains in this area.

For the treatment of gynaecological malignancies, pelvic angiography and embolisation techniques are similar to cases with PPH. However, due to the risk of recurrent haemorrhage, permanent embolic agents are strongly advised.

### Medication and peri-procedural care

Haemodynamic support is a prerequisite for OHE. Thus, dedicated anaesthetic input is required, including fluid management, administration of tranexamic acid, uterotonic drugs, red blood cell units (RBCUs), fibrinogen, fresh frozen plasma (FFP), platelets (PLT), and activated recombinant human factor seven (rhFVIIa), particularly in cases of acute haemorrhage to simultaneously resuscitate the patient and ensure patient comfort so embolisation can be performed swiftly.

### Post-procedural follow-up care

Repeat bleeding after OHE for obstetric haemorrhage is seen in 5–10% of patients. Causes include arterial spasm, collateral vessels and invasive placenta in PPH. If uterine arteries are still patent, repeat OHE of uterine arteries is a primary option. In the case of clear occlusions of uterine arteries, or of continued bleeding despite uterine embolisation, other bleeding sources should be evaluated, such as collaterals or spontaneous anastomoses. Collateral vessels might occur from ovarian vessels, rectal, iliolumbar, lumbar or even mesenteric arteries, and in rare cases from the artery of round ligament. Prior cross-sectional imaging may be helpful in such cases, or proceeding to non-selective aortography from the level of the renal arteries.

Post-embolisation syndrome may follow embolisation and is characterised by pain, fever, nausea, and leukocytosis which can last several days. It is treated supportively with analgesic and anti-inflammatory medication.

## Results

### Outcomes and effectiveness

Although there are no large randomised studies, and the level of evidence is limited, it may be stated that arterial embolisation is a safe and effective procedure for PPH and gynaecological haemorrhage (Table 2) (Boulleret et al., 2004; Ornan et al., 2003; Deux et al., 2001; Ratnam et al., 2008; Tsang et al., 2004; Tourné et al., 2003; Maassen et al., 2009; Hong et al., 2004; Ojala et al., 2005; Spreu et al., 2017). For PPH, reported success rates range from 79 to 100% (Table 2). Factors predicting embolisation failure include accessory arterial blood supply, previous surgical ligation, dilatation and curettage, unilateral embolisation and the presence of abnormal placentation and also caesarean section.

**Table 2:** Table summarising technical success rates and complications from published series of trans-arterial embolisation for post-partum haemorrhage

Author	Year of publication	No. of patients	Technical success rate	Repeat embolisation required (n)	Major complications (n)
Boulleret et al.	2004	35	100%	9% (3)	11% (4)
Ornan et al.	2003	28	96%	0%	14% (4)
Pelage et al.	1998	27	93%	4% (1)	4% (1)
Deux et al.	2001	25	96%	8% (2)	0%
Ratnam et al.	2008	19	79%	0%	0%
Tsang et al	2004	12	100%	0%	0%
Tourne et al.	2003	12	92%	0%	0%
Maassen et al.	2009	11	82%	18% (2)	18% (2)
Hong et al.	2004	10	100%	0%	0%
Ojala et al.	2005	22	100%	0%	13% (3)
Spreu et al.	2017	16	100%	6% (1)	0%

### Complications

Overall complication rates for obstetric and gynaecologic OAEs are 4–18% (Table 2). They comprise general angiographic complications, such as groin puncture site hematoma, dissections and contrast medium reactions. Non-target embolisation is a rare event, but can lead to ovarian failure or necrosis of the bladder or rectum. Rarely, necrosis of the small bowel, uterus,vagina and labia have also been observed. Uterine necrosis requiring hysterectomy is extremely rare. Buttock ischemia and claudication is also a potential complication, that might appear as a transient and minor complication, but could be lifestyle-limiting. Such complications can arise from zealous over-embolisation, use of inappropriately small particles in cases of shunts, or interruption of collateral supply by previous ligations (Poujade et al. 2013).

Neurological complications are also very rare, but may occur due to communications between the IIA and arteries supplying the spinal cord and the sciatic and femoral nerves. Use of very small particles or non-selective liquid embolisation might cause such problems and should be avoided (Rohilla et al. 2014).

Transient ovarian failure after OHE is described, however, generally after OHE women can expect return to normal menses and fertility.

## Conclusions

OHE in the management of PPH is the preferred treatment in otherwise uncontrollable post-partum bleeding and should be done when primary medical therapy failed. It should be considered before surgical therapy is instituted and should be considered early enough in the management of a haemorrhaging patient.

Embolisation has also been proven as an effective procedure for bleeding control in other gynaecological conditions, such as bleeding from tumours and AVMs.

Transcatheter embolisation is a safe and successful method if performed accordingly.

## Data Availability

Not applicable.

## Abbreviations

OHE: Obstetric haemorrhage embolisation
PPH: Postpartum haemorrhage
AVMs: Arteriovenous malformations
AV: Arteriovenous
IIA: Internal iliac artery
UA: Uterine artery
UAE: Uterine artery embolisation
MR-A: MR-Angiography
DSA: Digital subtraction angiography
RBCUs: Red blood cell units
FFP: Fresh frozen plasma
PLT: Platelets
rhFVIIa: Activated recombinant human factor seven
